# Supplementary material for: Early maturation and hyperexcitability is a shared phenotype of cortical neurons derived from different ASD-associated mutations
Source: Transl Psychiatry. 2023 Jul 6;13:246. doi: 10.1038/s41398-023-02535-x (PMC10326262; doi:10.1038/s41398-023-02535-x)

a

Cell lines' ID: UOHi011_O_Dup7_NPC, UOHp011 _Dup7_PBMCs

Marker	UOHi011_O_Dup7_NPC	UOHp011 _Dup7_PBMCs
AMEL	X,Y	X,Y
D3S1358	16,18	16,18
D1S1656	15,17	15,17
D2S441	11	11
D10S1248	15,16	15,16
D13S317	11,12	11,12
Penta E	7,10	7,10
D16S539	9,11	9,11
D18S51	13,15	13,15
D2S1338	20,24	20,24
CSF1PO	11,12	11,12
Penta D	10,11	10,11
TH01	6,9.3	6,9.3
vWA	17	17
D21S11	28,32.2	28,32.2
D7S820	9,10	9,10
D5S818	11	11
TPOX	8	8
DYS391	10	10
D8S1179	14,16	14,16
D12S391	19,22	19,22
D19S433	13.2,15	13.2,15
FGA	22	22
D22S1045	15,16	15,16

Summary

The samples profiles show that lines UOHi011_O_Dup7_NPC and UOHp011 _Dup7_PBMCs completely match one another.

b

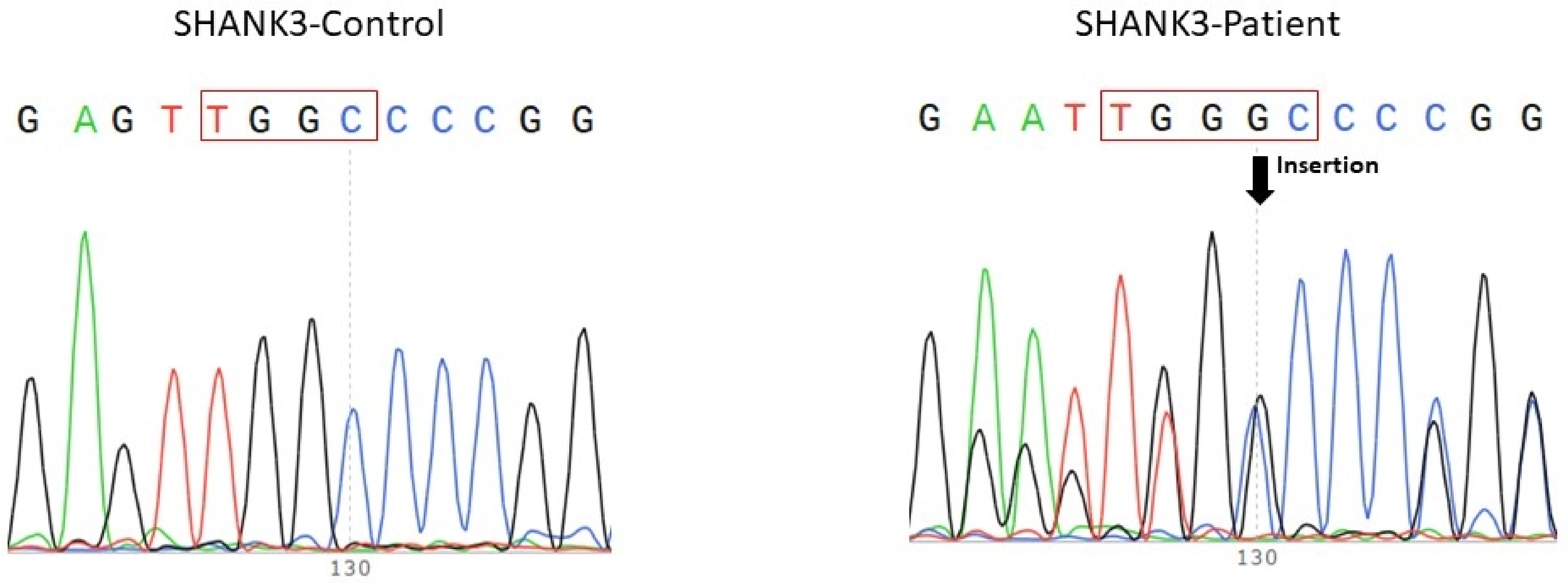

Supplement: Supplementary file 1 — Figure S1 [file 41398_2023_2535_MOESM1_ESM.pdf]
