# Supplementary material for: Early maturation and hyperexcitability is a shared phenotype of cortical neurons derived from different ASD-associated mutations
Source: Transl Psychiatry. 2023 Jul 6;13:246. doi: 10.1038/s41398-023-02535-x (PMC10326262; doi:10.1038/s41398-023-02535-x)
Supplement: Supplementary file 2 — Supplementary Information [file 41398_2023_2535_MOESM2_ESM.docx]

**Fig S1. STR and PCR tests validate the existence of the mutations in Dup7 and *SHANK3*.**

**a** The sample profiles show that the PBMC and NPC lines of UOHi011 completely match one another. **b-c** PCR validation of the (C.3679insG) *shank3* mutation in the mutant line and not in the control line.

**Table T1.** A description of the patients' cohort

| **Patient ID** | **Mutation** | **Sex** | **Age at sampling (Y)** | **Clinical symptoms** |
| --- | --- | --- | --- | --- |
| UOHi011 | Dup7 (7q11.23 dup) | M | 7.5 | ASD, minor ID |
| UOHi010 | Control | M | 39 | - |
| 784313 | GRIN2B (c.2065 G->T) | F | 2 | ASD, ID |
| UOHi010 | Control | M | 39 | - |
| UOHi003-A | SHANK3 (C.3679insG) | F | 10 | ASD, ID |
| UOHi002-A | Control | F | 47 | - |
| UOHi004 | UBTF (E210K) | F | 22 | ASD, ID |
| UOHi005 | Control | F | 50 | - |

**Table T2.** Spike shape analysis (non-parametric statistical tests: Wilcoxon signed rank test) of mutant and control cortical neurons.

|  | **Dup7** | **GRIN2B** | **SHANK3** | **UBTF** |
| --- | --- | --- | --- | --- |
| **Spike Threshold** | µ_mutant_ =27.6 + 6.5 mV  µ_Ctl_ =21.09 + 12.05 mV | µ_mutant_=21.5 + 3.6 mV  µ_Ctl_=22.2 + 6.7 mV | µ_mutant_=30.4 + 5.6 mV  µ_Ctl_=25.5 + 4.2 mV | µ_mutant_=32.1 + 4.9 mV  µ_Ctl_=21.5 + 16.7 mV |
|  | P = 0.29 | P = 0.27 | **P = 0.02 *** | P = 0.07 |
| **Spike Height** | µ_mutant_=25.4 + 15.5 mV  µ_Ctl_=27.6 + 19.4 mV | µ_mutant_=41.2 + 12.5 mV  µ_Ctl_=20.5 + 14.01 mV | µ_mutant_=44.3 + 11.4 mV  µ_Ctl_=34.4 + 7.5 mV | µ_mutant_=50 + 17.05mV  µ_Ctl_=36.04 + 18.01 mV |
|  | P = 0.14 | **P = 0.008 **** | P = 0.09 | **P = 0.03 *** |
| **Spike width** | µ_mutant_ =3.2 + 3.4 ms  µ_Ctl_ =2.4 + 3.4 ms | µ_mutant_=8.3 + 5.8 ms  µ_Ctl_=27.6 + 19.4 ms | µ_mutant_=6.5 + 5.9 ms  µ_Ctl_=5.9 + 1.2 ms | µ_mutant_=3.3 + 1.5 ms  µ_Ctl_=12.5 + 17.8 ms |
|  | P = 0.7 | P = 0.065 | P = 0.17 | **P = 0.003 **** |
| **Rise time** | µ_mutant_ =1.9 + 3.1 ms  µ_Ctl_ =0.68 + 0.87 ms | µ_mutant_=7.2 + 5.1 ms  µ_Ctl_=3.6 + 1.9 ms | µ_mutant_=4.6 + 2.6 ms  µ_Ctl_=4.6 + 1.8 ms | µ_mutant_=2.5 + 1.6 ms  µ_Ctl_=5.2 + 5.3 ms |
|  | P = 0.34 | P = 0.23 | P = 0.58 | P = 0.18 |

*P<0.05, **P<0.01, ***P<0.001 and ****P<0.0001
